# ASSOCIATIONS OF GAIT CHARACTERISTICS AND VARIABILITY WITH WALKING EFFICIENCY IN OLDER ADULTS

**DOI:** 10.1093/geroni/igac059.1194

**Published:** 2022-12-20

**Authors:** Fangyu Liu, Yurun Cai, Laura Skow, Amal Wanigatunga, Qu Tian, Eleanor Simonsick, Jennifer Schrack

**Affiliations:** Johns Hopkins Bloomberg School of Public Health, Baltimore, Maryland, United States; University of Pittsburgh, Pittsburgh, Pennsylvania, United States; Johns Hopkins Bloomberg School of Public Health, Baltimore, Maryland, United States; Johns Hopkins Bloomberg School of Public Health, Baltimore, Maryland, United States; National Institutes on Aging, Baltimore, Maryland, United States; National Institute on Aging, Baltimore, Maryland, United States; Johns Hopkins Bloomberg School of Public Health, Baltimore, Maryland, United States

## Abstract

Higher energetic cost of walking per unit distance has been linked to many adverse health outcomes in older adults. Aging-related changes in gait characteristics have been postulated to contribute to energetic inefficiency, but previous studies focused on younger adults, and/or examined only a few gait characteristics. In the Baltimore Longitudinal Study of Aging, 507 older adults ≥50 years (72.3±9.8 years, 48.3% women, 48.3% black) without stroke and Parkinson disease had concurrent measurements of usual-paced gait characteristics using 3D motion analysis and energetic cost of walking using indirect calorimetry during a 2.5-min usual-paced overground walk. We tested the associations of the mean and the coefficient of variance (CV) of cadence (steps/min), swing time (ms), double support time (ms), stance time (ms), step time (ms), step length (cm), and step width (cm) with energetic cost of walking using linear regression models, adjusting for demographics, body composition, comorbidities, and gait speed. We found that a 5-cm shorter step length was associated with 0.40 ml/kg/100m higher cost of walking (p< 0.001). A 1% higher CV in swing time and 1% lower CV in step width was associated with 0.152 ml/kg/100m higher (p=0.044) and 0.023 ml/kg/100m lower (p=0.022) cost of walking, respectively. Our results suggest that mean step length and variability in swing time and step width could potentially contribute to the rising cost of walking in older adults. Future longitudinal studies are needed to understand whether changes in gait variability can predict increased energetic cost of walking and can be intervened to preserve energy efficiency.

